# Basigin (CD147) and calpain 4 (CapnS1) are partners in the generation of traction force but not in mechanosensing

**DOI:** 10.1016/j.jbc.2026.113170

**Published:** 2026-05-18

**Authors:** Jacob DeTone, Savannah Kozole, Mia Stewart, Bingqing Hao, Karen A. Beningo

**Affiliations:** Department of Biological Sciences, Wayne State University, Detroit, Michigan, USA

**Keywords:** calpain, basigin, migration, traction stress, mechanosensing, glycosylation, cancer

## Abstract

Traction force and mechanosensing (the ability to sense the mechanical attributes of the environment) are two key factors that enable a cell to modify its behavior during migration. Previously, it was determined that the calpain small subunit, Calpain 4 (CapnS1), regulates traction force production independent of its proteolytic holoenzyme. A proteolytic enzyme is formed by Calpain 4 binding to either of its catalytic partners, Calpain 1 and 2. To further understand how Calpain 4 regulates traction force, we used two-hybrid analysis to identify more components of the traction pathway. We discovered that Basigin, an integral membrane protein and a documented inducer of matrix metalloproteases (MMPs), interacts with Calpain 4 in two-hybrid and pull-down assays. Traction force was reduced when basigin was silenced in MEF cells, and this reduction was also reflected in a similar defect in adhesion formation and in substrate adhesion strength. Unlike Capn4^−/−^ MEF cells, Basigin-deficient cells exhibited normal mechanosensing. Together, these results implicate Basigin in the pathway in which Calpain 4 regulates traction force independent of the catalytic large subunits.

Cell migration is essential for various physiological processes, both normal and abnormal, including embryonic development, wound healing, immune responses, and cancer metastasis. Cell migration is also significant to technological applications such as tissue engineering (1, 2, 3, 4). Although numerous studies have been conducted to extend our understanding of how the complex process of cell migration is regulated, the mechanism remains to be fully defined.

Focal adhesions function dynamically in cell migration, specifically in biophysical terms of transmitting both traction forces and mechanosensing between the actin cytoskeleton and extracellular matrix (ECM) (5, 6, 7, 8). Calpains are involved in cell migration as calpain proteases localize to focal adhesions and proteolyze the turnover of several focal adhesion components (9, 10, 11, 12). The two best-characterized calpains, μ-calpain and m-calpain, both contain a distinct 80 kDa catalytic large subunit (calpain 1/CAPN1 and calpain 2/CAPN2, encoded by *Capn1 and Capn2* genes, respectively) and a common 28 kDa small subunit (calpain 4/CAPNS1/CAPN4, encoded by *Capn4* gene) (12). Inhibiting Calpains through overexpression of the endogenous inhibitor Calpastatin and pharmacological inhibitors reduces both adhesive complex disassembly and actinin localization to focal contacts (10).

Calpains are known to be regulated post-translationally through phosphorylation, endogenous inhibitors, and interactions with the regulatory small subunit. The protein phosphatase 2A (PP2A) is identified as a Calpain phosphatase of μ-calpain and m-calpain and can directly dephosphorylate both heavy chains. PP2A dephosphorylation inactivates μ-calpain and m-calpain, thereby suppressing migration in lung cancer cells (13) (Xu and Deng, 2006). The small subunit was historically considered to mainly serve a regulatory function for calpain holoenzymes (12). However, a finding that *Capn4*^*−/−*^ embryonic fibroblasts exhibit abnormal organization of focal adhesions, reduced rates of cell migration, and delayed retraction of membrane projections implicates the small subunit in regulating cell migration (11). In addition, we found that traction force was attenuated by calpain small subunit knockout or knockdown, but not by knockout or knockdown of the large subunits. In contrast, all subunits are required for mechanosensing. Our previous study implicated the small subunit in regulating traction force as an entity independent of the large subunits (14, 15).

To gain insight into how the calpain small subunit regulates traction force production, we screened for binding partners. In this current study, we used the entire Capn4 gene as bait in a yeast two-hybrid assay. From a screen of the whole mouse embryonic genome, we identified the protein Basigin as a potential binding partner of Calpain 4 amongst a limited number of candidates.

Basigin (*Bsg*), also known as CD147 or EMMPRIN, is a heavily glycosylated transmembrane protein belonging to the immunoglobulin (Ig) superfamily (16, 17). Basigin has been found to function in various cellular processes and in cancer progression. Mice deficient in the basigin gene have abnormal embryogenesis, spermatogenesis, and fertilization (18, 19, 20). Knockout mice of the *Bsg* gene display abnormalities in vision and insensitivity to irritating odor (21, 22). Basigin is commonly overexpressed in many tumors (23, 24, 25) and is associated with almost all types of cancer (24). On the surface of tumor cells, Basigin was found to stimulate the production of matrix metalloproteinases (MMPs) in adjacent fibroblasts, thereby enhancing tumor invasion and leading to the name EMMPRIN (26, 27).

Basigin is known to interact (directly or indirectly) with several proteins, including MCT1, MCT2, Integrin-β1, Cyclophilin, Caveolin-1, Annexin-2, and Ubiquitin C (24, 28, 29, 30). Many of its known interacting proteins are implicated in cell migration and secretion. Basigin’s functions in tumorigenesis and cell migration render it a reasonable target for elucidating how calpain 4 regulates traction force production.

In this study, Basigin was identified as a binding partner of Calpain 4 using the yeast two-hybrid assay, subjected to predictive analysis by AlphaFold Server, and subsequently verified by immunoprecipitation. Furthermore, we found that upon Basigin knockdown, traction force was significantly reduced, and these cells were defective in substrate adhesion. Surprisingly, MEFs in which Basigin is silenced can respond to localized stimuli and sense the stiffness of substrates, like wild-type cells, suggesting that Basigin functions only in the production of traction stress with Calpain 4, not in mechanosensing, where Calpain 4 is needed. These results implicate Basigin in the same pathway that Calpain 4 uses to regulate the production of traction force, a pathway independent of the holoenzyme's catalytic activity.

## Results

### Basigin as a binding partner of Calpain 4

**Figure 1:**
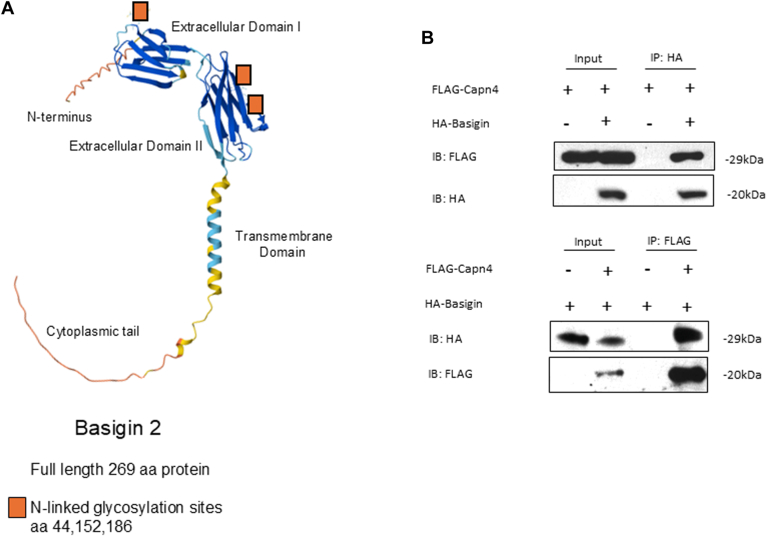
**Co-immunoprecipitation to confirm the interaction between Calpain4 and Basigin proteins.***A*, illustration of the transmembrane protein Basigin 2 (modified AlphaFold predicted structure). *B*, the entire gene of *Capn4* was inserted into a pFLAG-CMV vector, and the Bsg gene, lacking a 100 amino acid sequence for the N-terminal, was inserted into a pCDNA3 vector together with an HA sequence. Both plasmids were transfected into 293T cells. Pull-down assays were performed using either a FLAG or an HA antibody to confirm direct binding between Calpain 4 and Basigin.

To further elucidate the mechanism by which calpain4 produces traction force, the full Capn4 gene was inserted into the plasmids pCWX200 and pLexA and used as bait to identify binding partners for the Calpain 4 protein in a yeast two-hybrid assay. Sequencing identified Basigin as a candidate binding partner for Calpain 4 (Table 1). Additionally, all proteins identified in the yeast two-hybrid assay were submitted to the AlphaFold Server to assess the likelihood of true interaction. Syndecan-1 and Basigin were the most likely physiological candidates, but Basigin’s AlphaFold Server score was strikingly low, due to a limitation in the software as compared to more structurally stable proteins like Capn1 (Table 1). To better assay interaction with Basigin, we used co-immunoprecipitation. This interaction between Calpain 4 and Basigin (less 100 a.a. of the n-terminus) was confirmed by co-immunoprecipitation (Fig. 1 *B*). The interaction between Calpain 4 and Basigin suggests that Basigin is involved in the pathway that generates traction forces regulated by Calpain 4.

**Table 1:** Candidate-Binding Partners for Capns1 isolated from Yeast-Two-Hybrid

Binding partners	Full name	Function	AlphaFold server iPTM/PTM score
Capns1	Calpain small subunit 1	A shared small subunit to both Capn1 and Capn2 (54)	0.66/0.66
Vps24	Vacuolar protein sorting 24	Protein sorting *via* multivesicular body pathway (55, 56)	0.11/0.42
Ivns1abp	Influenza virus NS1A binding protein	Splicing of NS1-BP pre-mRNA (57, 58)	0.21/0.41
Capn1	Calpain 1	Large subunit of the calpain holoenzyme, calpain 1 (59)	0.76/0.76
Sdc1	Syndecan 1	Cell proliferation, migration, and cell-matrix interaction (60, 61)	0.62/0.38
Nr1h2	Nuclear receptor	Macrophage inflammation & lipid homeostasis (62)	0.61/0.51
Zbtb11	Zinc finger and BTB domain containing 11	Transcriptional regulation (63)	0.31/0.25
Npy	Neuropeptide Y	Neurotransmitter (64)	0.31/0.56
Tmem176b	Transmembrane protein 176B	Dendritic cell maturation (65)	0.16/0.39
Bsg	Basigin	Various immunological phenomena, differentiation, development, including tumor invasion (16, 27)	0.09/0.36∗

Proteins identified from yeast two-hybrid screening using full-length Capn4 as bait are listed with their full names, brief functional descriptions, and literature citations. To assess the structural plausibility of these interactions, each candidate protein sequence was submitted to the AlphaFold multimer prediction server together with Capn4, and the resulting interaction confidence metrics are reported as iPTM/PTM scores. The interface predicted TM-score (iPTM) estimates the confidence of the predicted protein–protein interface, whereas the predicted TM-score (PTM) reflects the overall structural accuracy of the multimeric model. Scores range from 0 to 1, with higher values indicating greater model confidence and a higher likelihood of a stable interaction interface. An asterisk denotes the Basigin prediction, which shows a low iPTM score; this likely reflects the intrinsically disordered nature of the Basigin cytoplasmic tail, which can reduce AlphaFold multimer confidence despite the possibility of biologically relevant interactions.

**Figure 2:**
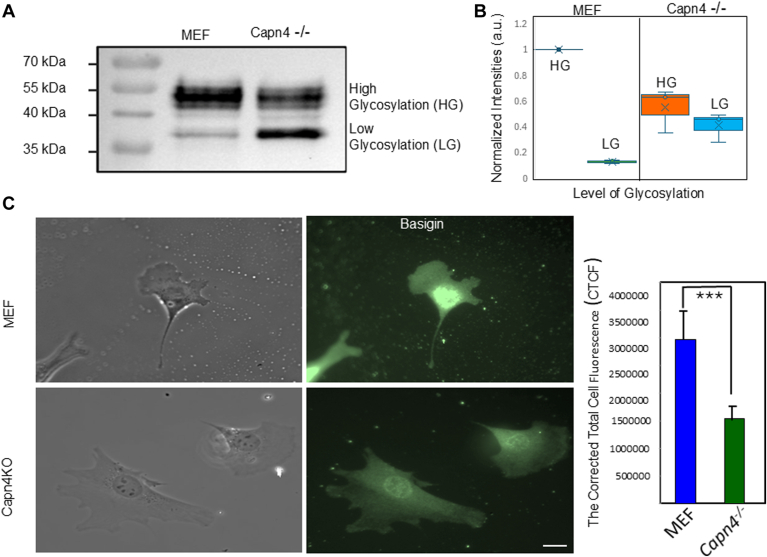
**Basigin expression level is reduced in *Capn4−/−* cells.***A*, two bands of 50 and 37 kDa were detected in cell lysates by Western blot probed with monoclonal anti-basigin. The bands represent high and low levels of Basigin glycosylation, respectively. *B*, intensities normalized to total protein (Material S1*A*), and glycosylation confirmed (Material S1*B*). *C*, localization of Basigin in MEFs and *Capn4−/−* cells. Cells were seeded onto fibronectin-coated coverslips. ImageJ calculated the corrected total cell fluorescence (CTCF) for Basigin in each cell line. *Capn4*^*−/−*^ cells exhibit significantly reduced levels of CTCF when compared to MEF (*p* = 0.003). 12 cells were used per cell line for the calculation. Data for *A* and *B*, represent three independent trials. (Mag. bar = 20 μm).

When comparing the endogenous expression level of Basigin in both MEFs and *Capn4−/−* cells, we observed that Basigin’s glycosylation was altered in *Capn4−/−* cells compared to MEFs (Fig. 2*A*), as supported by quantification (Fig. 2*B*) (Supporting Information 1*A*). Basigin is known to be glycosylated at three asparagines (Fig. 1*A*). The glycosylation state can result in multiple bands and is referred to as high- and low-glycosylation states. Glycosylation can be demonstrated by enzyme digestion with PNGase F (Supporting Information 1*B*). Interestingly, in the absence of Calpain4, a lower glycosylation state of Basigin is observed in MEF cells (Fig. 2, *A* and *B*); however, the significance of these glycosylation states remains to be determined.

To observe the expression pattern of the Basigin protein and investigate potential co-localization with Calpain 4, immunofluorescence was performed in both MEFs and Capn4−/− cells using a Basigin antibody. Basigin is predominantly localized to the nuclear/perinuclear region and diffusely at the cell edge (Fig. 2*C*). Basigin does not strongly localize to the periphery of *Capn4−/−* cells. However, this result is likely due to the thinness of the lamellipodia in *Capn4−/−* cells, as previously described (14). Taken together, we find evidence of an interaction between Basigin and Calpain 4 *via* two-hybrid and co-immunoprecipitation assays, as well as differences in Basigin glycosylation and cellular immunofluorescence in *Capn4−/−* cells.

### Knockdown of basigin resulted in defects in traction force, adhesion strength and abnormal adhesion localization in MEFs

Previous studies on cell migration revealed a deficiency in traction forces in Capn4−/− cells compared with wild-type MEF cells, whereas inhibition of Capn1 or Capn2, or overexpression of calpastatin, did not affect traction forces (14, 15). Given the interaction between Basigin and Calpain 4, we knocked down Basigin and assessed whether this affected traction force production. Basigin was knocked down by nucleofection of siRNA in MEF cells, resulting in a high efficiency (95%) of inhibition after 36 h, as demonstrated by western blots and quantification (Supporting Information 2).

**Figure 3:**
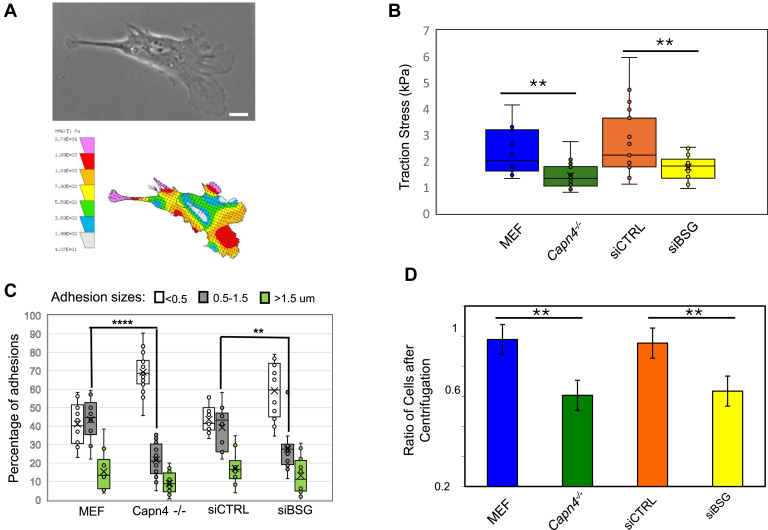
**Basigin traction stress and adhesion strength mimics Capn4 −/−.***A*, a phase image representing an MEF cell nucleofected with siBSG and seeded on fibronectin-coated polyacrylamide substrates overnight before TFM, and a color map of the traction stress of the cell in the image of *panel A.* The map depicts warm colors as high stress and cool colors as low stress. The *arrows* indicate the magnitude, location, and direction of the stress. The strongest stress is found at the leading edge, consistent with previously reported *Capn4−/−* TFM. *B*, A graph representing the average traction stress exerted by each cell line onto the substrate. Traction stress of Basigin knock-down MEFs was compared with MEFs, *Capn4*^*−/−*^ cells, and MEFs transfected with control siRNA. When Basigin was silenced by siRNA, the traction stress level was significantly reduced, as well as in *Capn4*^*−/−*^ cells, compared to MEFs and MEFs transfected with control siRNA. 11 to 15 cells from each cell line were used to calculate traction forces. *C*, Box-and-whisker plot of adhesion for wild-type MEFs, *Capn4−/−* cells, MEFs nucleofected with control siRNA, and MEFs nucleofected with Basigin siRNA. 10 MEFs, 20 *Capn4−/−* cells, 10 MEFs nucleofected with control siRNA, and 12 MEFs nucleofected with Basigin siRNA were used for adhesion quantification within three size ranges (<0.5 μm^2^, 0.5–1.5 μm^2^, >1.5 μm^2^). A total of 1422 adhesions were counted. *D*, A bar graph representing the adhesion strength of cells by calculating the percentage of the number of cells that remained adhered onto the substrates after centrifugation and compared to MEFs, *Capn4*^*−/−*^ cells exhibited significantly reduced adhesion strength (*p* = 0.02). When basigin was silenced through siRNA in MEFs, a reduction of adhesion strength was also observed (*p* = 0.02). Three independent experiments are represented. A two-tailed Student’s *t* test was performed to analyze the statistical significance.

To measure the traction stress generated by the Basigin knockdown, cells were seeded onto a flexible polyacrylamide substrate covalently coated with fibronectin and analyzed by traction force microscopy (TFM) (31). Traction stress in each cell line was measured (Fig. 3, *A* and *B*). As expected, *Capn4*^*−/−*^ cells produced significantly less traction stress (avg. 1.5 kPa) compared to wild-type MEFs (avg. 2.69 kPa, *p* = 0.03) and MEFs transfected with control siRNA (avg. 2.91 kPa, *p* = 0.04). Furthermore, silencing Basigin in MEFs significantly reduced the magnitude of traction forces to 1.63 kPa (*p* = 0.04) (Fig. 3, *A* and *B*). These results demonstrate that silencing basigin leads to a deficiency in traction stress production comparable to that observed upon disruption of calpain 4.

We have previously documented an atypical localization of adhesions in *Capn4−/−* cells. These knockout cells had fewer adhesions, measuring 0.5 to 1.5 um in length, often clustered along the cell edge, indicative of an adhesion maturation defect. Given the defects in strength and traction, we tested whether Basigin knockdown cells also exhibit the same localization defect. We stained for vinculin and binned adhesions by length (<0.5, 0.5–1.5, >1.5 μm) in MEF, Capn4−/−, siCntrl, and siBsg that were incubated on fibronectin-coated coverslips. Capn4−/− and siBsg had significantly fewer adhesions of the 0.5 to 1.5 um length (*p*-value 0.0001and 0.03, respectively) when compared to their appropriate control (Fig. 3*C*).

To assess the adhesion strength of focal adhesions to substrates, we performed a centrifugation assay using the same set of cell lines as above (14, 15, 32). Cells were seeded onto fibronectin-coated flexible polyacrylamide substrates and allowed to adhere for 30 min at 37 °C, then centrifuged. The number of cells adhered to the substrates was counted before and after centrifugation. We found that approximately 61% of *Capn4−/−* cells remained adhered to the substrates after centrifugation, compared to 98% of MEFs that remained adhered (*p* = 0.02) (Fig. 3*D*). Similarly, silencing Basigin resulted in only approximately 63% of cells remaining adhered to the substrates (*p* = 0.02) (Fig. 3*D*). In comparison, 95% percent of MEFs treated with control siRNA remained adhered after centrifugation (Fig. 3*D*).

These results suggest that Basigin contributes to adhesion maturation and adhesion strength, in addition to regulating the production of traction force, consistent with the behavior observed in *Capn4−/−*.

### Knockdown of basigin does not influence the mechanosensing of localized stimuli

Cells can sense mechanical information from the environment, including matrix elasticity, localized mechanical forces, and topography (33, 34, 35, 36, 37). These physical signals are transmitted from the outside in and lead to changes in cytoskeletal networks, interactions with the extracellular matrix (ECM), cellular force production, differentiation, growth, and apoptosis (33, 34, 36, 37, 38). Our previous studies found that MEFs respond to localized mechanical stimulation by changing their migration trajectory or rounding up. However, when cells deficient in either Calpain 1, 2, or 4 are tested in this assay, they continue to migrate along the same trajectory, indicating they are insensitive to localized stimuli (14, 15). In another assay comparing the ability of cells to spread on substrates of different stiffness, the results indicate that MEFs can sense stiffness by spreading more completely on stiff substrates compared to soft substrates (15, 39). Surprisingly, MEF cells deficient in any calpain 1, 2, or 4 are still able to sense the stiffness difference and spread differently on hard and soft substrates (14, 15). Traction forces were believed not only to drive cell migration but also to play equal roles in sensing the physical environment (33). As our study indicated that silencing Basigin in MEFs significantly affected the generation of traction forces, we tested Basigin knockdown cells for their ability to sense changes in both mechanosensing assays.

**Figure 4:**
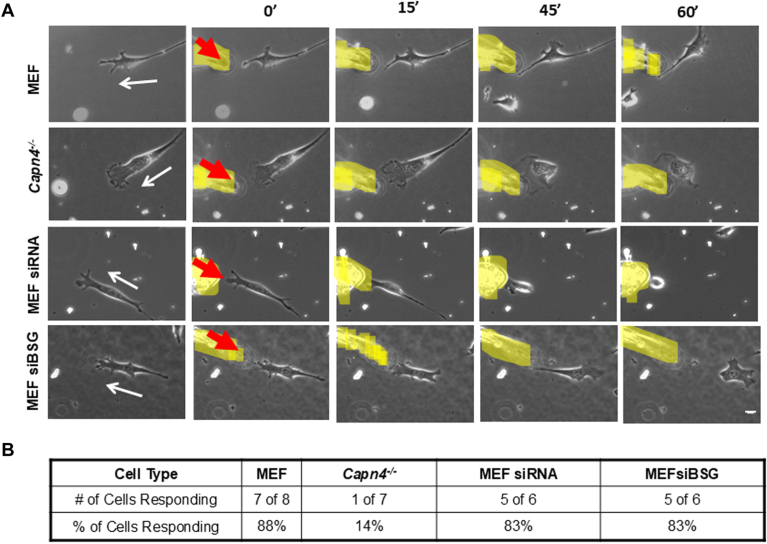
**Silencing Basigin does not affect the ability of cells to sense changes in the mechanical environment.***A*, representative time-lapse images illustrate the responses of cells to a localized stimulus, including MEFs, *Capn4*^*−/−*^ cells, MEFs transfected with control siRNA, and Basigin knockdown MEFs. The thin white arrows in the first column denote the original cell migration direction; the *bold red arrows* in the second column denote the direction in which the blunted microneedle pushes on the substrate, thereby making the substrate softer in front of the migrating cell. The blunted microneedle is highlighted in *yellow*. Cells were seeded onto flexible polyacrylamide substrates that were covalently coated with fibronectin and allowed to attach overnight. A blunted needle was inserted into the substrate along the migration path, and the responses of cells were recorded for each cell line (magnification bar = 10 μm). The results were summarized in (*B*). The table indicates the percentage of cells of each cell line that respond to the localized change in the mechanical environment. The number of cells and percentage response for each cell line were summarized in the table. If a cell avoids the pushing force or rounds up, it is sensing and responding to the change; if a cell continues to migrate toward the pushing force, it is not sensing the localized change in stiffness.

Basigin knockdown cells were tested for their responsiveness to localized mechanical stimuli. In the assay, cells were seeded onto flexible polyacrylamide substrates coated with fibronectin, and a blunted needle was gently pushed onto the substrate against the direction of migration. As expected, 88% of MEF cells responded to the pushing force by avoiding it while only 14% of *Capn4*^*−/−*^ cells responded (Fig. 4, *A* and *B*). As with MEF controls, 84% of MEF cells transfected with control siRNA responded to the localized pushing force (Fig. 4, *A* and *B*). When Basigin was silenced in MEFs, 84% of cells still responded to localized force (Fig. 4, *A* and *B*). Our results indicate that Basigin does not play a role in sensing localized mechanical stimulus.

**Figure 5:**
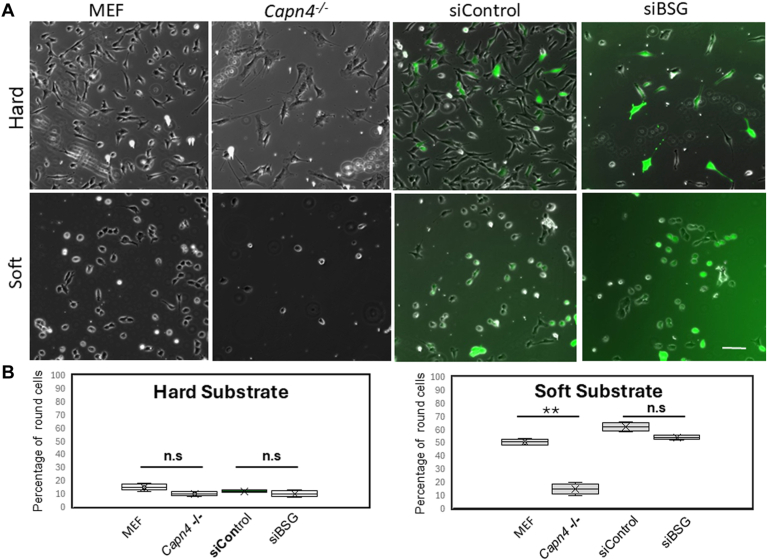
**Basigin-deficient cells can sense a soft environment, but C*apn4−/−* cells do not.***A*, images were taken with a 10× lens for each cell line after they were seeded on both hard (5%/0.1% Acryl/Bis, 1981 Pa) and soft (5%/0.04% Acryl/Bis, 330.2 Pa) substrates and allowed to adhere overnight. The number of round cells was then counted for each line as observed visually. *B*, the average cell counts for each line were graphed; six random fields were counted for each cell line. Statistical analysis was performed by Student’s *t* test. ∗ indicates *p* = 0.03.

To test whether cells deficient in Basigin can sense a more global, homeostatic change in the stiffness of the environment, cells were seeded onto hard and soft flexible polyacrylamide substrates coated with fibronectin and allowed to adhere overnight. Cells were scored for a rounded phenotype. As expected, when seeded on rigid substrates, 85% of MEFs spread normally on hard substrates, as well as 91% of *Capn4*^*−/−*^ cells. Meanwhile, 86% of MEFs treated with control non-target siRNA and 90% of MEFs treated with Basigin-targeting siRNA also spread normally (Fig. 5, *A* and *B*). In contrast, only 47% of MEFs, 85% of *Capn4*^*−/−*^ cells (*p* = 0.02), 37% of MEFs treated with control non-target siRNA, and 44% of MEFs treated with Basigin targeting siRNA spread well when seeded on soft substrates (*p* > 0.05) (Fig. 5, *A* and *B*). The significant decrease in the number of cells spreading normally on substrates of different stiffness indicated that Basigin is not involved in sensing the stiffness of the environment. Combined with the cellular responses to a localized stimulus, unlike Calpain-4, Basigin is not significant to the mechanosensing process.

### Knockdown of basigin does not affect cell migration speed and migration persistence

The speed and persistence of cell migration are affected by multiple factors, including dimension, matrix stiffness, cell–cell and cell–matrix adhesion, traction forces, cytoskeletal polarity, and the capacity to degrade ECM by proteolytic enzymes (40, 41). Previous observations indicate that *Capn4−/−* cells have a reduced migration speed compared to MEF cells (11, 14) when cultured on fibronectin-coated coverslips; however, this difference is not observed on fibronectin-coated polyacrylamide substrates (15). To assess whether inhibition of Basigin affected cell migration, Basigin knockdown cells were seeded onto fibronectin-coated polyacrylamide substrates and observed for 2 h to track nuclear locomotion. Consistent with previous studies, *Capn4*^*−/−*^ cells migrated at linear speeds of (0.45 μm/min) compared to MEFs (0.48 μm/min, *p* = 0.60). When Basigin was silenced, an insignificant difference in linear speed was also observed, such that MEFs silenced with siBSG migrated at 0.47 μm/min, (*p* = 0.65) on fibronectin-coated polyacrylamide substrates (Fig. S3*A*). As with migration speed, silencing Basigin did not affect migration persistence and was similar in all lines (Fig S3*B*). These results demonstrate that the Calpain 4 mechanism, which also involves Basigin and regulates traction generation, does not affect linear migration speed or persistence.

### Correlation of gene expression of CAPN4 and BSG in various human tumors

**Figure 6:**
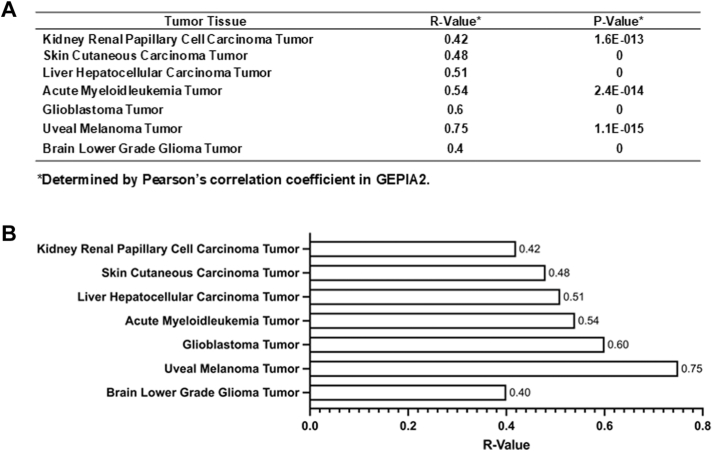
**R and *p*-Values for CAPN4 and BSG Expression Correlation in Various Tumor Tissues.***A*, Pearson’s correlation analysis was performed using GEPIA2 (Tang *et al.*, 2019), which enabled the modeling of CAPN4 and BSG expression across seven tumor tissues sourced in the TCGA database. From the Correlational Analysis function of GEPIA2, R and *p*-values, determined by Pearson’s correlation coefficient, for each of the respective tissues were collected and modeled in a table. The R-value quantifies the strength and linearity of the correlation between CAPN4 and BSG expression: an R-value greater than 0.5 indicates a strong, positive relationship, and a value between 0.3 and 0.5 indicates a moderate, positive relationship. Further, the *p*-value validates statistical significance. Three reported correlations were statistically significant (*p* < 0.001). *B*, a bar graph showing the R-values for the correlation between CAPN4 and BSG expression in seven tumors of interest.

In previous studies, both BSG and CAPN4 were independently implicated in tumor progression (42, 43). Based on this knowledge, we investigated whether their expression was correlated across an array of tumors. Using the GEPIA2 platform, which integrates gene expression data from the GTEx and TCGA databases, Pearson’s correlation coefficients were computed to assess the correlation between CAPN4 and BSG expression in several tumors (Fig. 6*A*) (44). Amongst these tumors, R-values ranged from moderate (R > 0.30) to strong (R > 0.50), and all correlations were considered positive; however, they vary in statistical significance (*p* < 0.001). The strongest correlation was observed in Uveal Melanoma (R = 0.75, *p* = 1.1 × 10ˆ (−15)) and Acute myeloid leukemia (R = 0.54, *p* = 2.4 × 10ˆ (−14)) tumors, indicating co-expression of BSG and CAPN4 in these malignancies. Furthermore, the most moderate correlation was observed in tumors of Kidney Renal Papillary Cell Carcinoma (R = 0.42, *p* = 1.6E-013) (Fig. 6*B*). Of particular interest is the shared neural origin and aggressive nature of Uveal Melanoma and Glioblastoma, suggesting that the co-expression of BSG and CAPN4 may contribute more significantly to tumor aggressiveness in cancers of neural origin. Collectively, these results indicate that, although the strength of correlation varies, the broader presence of CAPN4 and BSG correlative expression across diverse tumor tissues may be associated with enhanced tumor progression and cancer progression.

## Discussion

Our lab has previously discovered that Calpains participate in both the generation of traction stress and sensing localized stimuli in MEF cells (14). We found that both large and small subunits of Calpain holoenzymes are required for cells to normally sense localized stimuli, whereas only the small subunit is necessary to generate traction stress; however, silencing the large catalytic subunits has no effect on traction stress production (14). This suggests that the calpain small subunit functions independently of the large proteolytic subunits of Calpain in regulating traction forces, while all subunits are implicated in mechanosensing. In this study, we sought to gain insight into how this mechanism works by identifying direct binding partners of Calpain4.

We have discovered a previously unknown interaction between Basigin and Calpain 4, as determined by two-hybrid, AlphaFold Server, and co-immunoprecipitation assays. While AlphaFold Server’s predictive analysis contrasted with our two-hybrid and co-immunoprecipitation results, this technology has acknowledged limitations, including its inaccuracy in predicting transmembrane protein interactions and issues with intrinsically disordered regions (IDR). Specific to our comparisons, the cytoplasmic tail of Basigin, which we suspect binds Capn4, appears to be short and is flagged as an IDR. In addition, integral proteins are, in general, more difficult to model on AlphaFold due to a lack of membrane information. While it is possible to construct a “membrane mimetic” in software, modeling multi-protein interactions remains challenging. Even if one is to separate parts of Basigin (*e.g.*, Bsg cytoplasmic tail alone identified as an IDR, Bsg transmembrane domain in addition to cytoplasmic tail) to assay for interaction with Capn4 by AlphaFold Server, the interaction remains uncertain. For more structurally stable proteins, such as Capn1, we find a strong interaction score when paired with Calpain 4, serving as an internal positive control that is an established direct interaction in the literature. We also examined co-localization using immunofluorescence. We were not surprised to find a lack of co-localization between Basigin and Calpain4 *via* conventional immunofluorescence, given that Calpain4 localization is diffuse on its own. Qualitatively, it appeared that there were diminished signals for Basigin localization at the periphery of *Capn4−/−* cells. Basigin is known to have a diverse localization pattern, often dependent on the cell type. As expected, it frequently localizes to the plasma membrane; interestingly, it can also be found in focal adhesions, amongst other cellular locations, although we did not observe any localization. The high expression level and multiple functions of Basigin across diverse cell types likely explain the lack of localization observed in our study (Fig. 2*C*); however, more sophisticated microscopic analysis may yield further insight in future studies (18, 19, 20, 21, 22).

Studies of Basigin in cell migration have focused on its function in tumor cell motility and invasion. It has been reported that Basigin expression is elevated in most tumor cell types and is among the most highly expressed proteins in disseminated cancer cells (24). A high level of Basigin expression on the surface of tumor cells increases MMP activity in both stromal cells and tumor cells themselves (17, 45, 46). Elevated MMP activity degrades the ECM and alters ECM turnover dynamics, potentially leading to tumor cell motility and invasion (24). Consistent with these observations, our current study found that inhibiting Basigin expression through siRNA in wild-type MEFs results in reduced traction force and defective adhesion maturation and strength (Fig. 3); however, cells had normal migration speed (Fig. S3*A*), mirroring the Calpain 4 knockout and suggesting that Calpain 4 functions with Basigin in this pathway. However, Basigin affects numerous targets in addition to MMPs (17, 24). Other proteins are likely involved in regulating traction stress through this pathway. Further studies are needed to identify the components that function downstream of this signaling pathway.

Previous research indicates a deficiency in traction force in *Capn4−/−* cells (Fig. 3, *A* and *B*). Further, these *Capn4−/−* cells do not respond to localized stimuli and do not sense the stiffness of substrates normally (Figs. 4*A* and 5*A*), suggesting that Calpain 4 provides a means to separate traction force and mechanosensing spatially and temporally. Moreover, MEFs in which Basigin is silenced can respond normally to localized stimuli and sense substrate stiffness (Figs. 4*A* and 5*D*), suggesting that Basigin functions only in the production of traction forces, not in mechanosensing. Previous studies indicate that substrate rigidity sensing is driven by traction forces at the cell's leading edge, as localized softening of the frontal region results in cell retraction, reversal of cell polarity, or cell immobilization (47). Our conclusion does not contradict this observation, as we measured only the overall level of traction stress within a cell, not localized areas.

The difference in the glycosylation state of Basigin in Calpain-4-deficient cells was unexpected, though not without precedent. It is well documented that glycosylation is sensitive to various culture conditions, including temperature, pH, media composition, and cell passage (48). However, our observation of a lower glycosylation state of Basigin in *Capn4*^−/−^ cells remains consistent. There is a precedent that the degree of Basigin’s glycosylation can regulate its function (49). In our study, we speculate that it modulates Galectin-3 binding, a lectin that facilitates the clustering and activation of both Basigin and Integrin β1 (50). We have previously shown that Galectin-3 secretion requires Calpain 4, and that exogenous Galectin-3 can rescue the traction force defect (15). Although this mechanism is beyond the scope of the present study, it is currently under further investigation.

Basigin has been reported to regulate Integrin β1 *via* direct interaction; however, the literature presents conflicting evidence. In *Drosophila* perineurial glia, Basigin has been described as a negative regulator, while in other systems, it functions as a positive regulator (49, 51). Given that Galectin-3 promotes the clustering of Basigin and Integrin β1, and that Galectin-3-secretion-deficient mutants (Gal3 Y107F) exhibit reduced levels of active Integrin β1, it is plausible that Basigin serves as an activator of Integrin β1 in this context (15). The interplay between Basigin’s glycosylation state, its lateral organization at the membrane, Galectin-3 binding, and integrin β1 activation may constitute a dynamic regulatory axis underlying the generation of traction forces.

The link between Calpain4 and Basigin is intriguing and has left us questioning how often their expression correlates in cancers. As discussed, Basigin is known to affect cancer progression and to stimulate ECM degradation. Still, until now, the possibility of Calpain 4 being involved in cancer progression along with Basigin was unknown. Furthermore, the high correlation in neural cancers presents avenues for new research and potential therapeutic targets.

In summary, we have identified Basigin as a functional partner of Calpain 4. Further, we have determined that Basigin and Calpain 4 contribute to the regulation of traction force and substrate adhesion strength. However, Basigin is not implicated in mechanosensing and does not respond to localized stimuli and homeostatic tension. Together, these results associate Basigin with an unknown pathway in which Calpain 4 regulates traction force independently of the catalytic large subunits of Calpain 1 and 2.

## Experimental procedures

### Cell culture

Mouse embryonic fibroblasts expressing a defective small calpain subunit have been described previously (11, 52) and are referred to as *Capn4*^*−/−*^ cells in this study. Mouse embryonic fibroblasts (MEFs), *Capn4−/−* cells, and 293T cells were used in this study. MEFs were purchased from ATCC. 293T cells were a gift from Dr Xiangdong Zhang, University of Buffalo. MEFs and *Capn4*^*−/−*^ cells were maintained in Dulbecco’s Modified Eagle’s Medium-high glucose (Sigma) supplemented with 10% fetal bovine serum (FBS) (Hyclone) and 1% penicillin/streptomycin/glutamine (PSG, Gibco) and incubated at 37 °C under 5% CO2 in a humidified cell culture incubator. 293T cells were maintained and passed similarly, except that the media contained 1% PS instead of PSG. Cells were passaged by trypsinization using 0.1% trypsin-EDTA (0.25% trypsin-EDTA diluted in HBSS, Gibco) and then diluted and transferred into new culture dishes. Passages were limited to eight per cell line.

### Cloning of CAPN4 and yeast two-hybrid assay

Full-length *Capn4* was amplified by PCR from a pEGFP-capn4 plasmid under the following conditions: 30 cycles of 98 °C for 10 s followed by 68 °C for 1 min using PrimeSTAR HS DNA Polymerase with GC buffer (Takara, R044A). The primers used to insert *Capn4* into pCWX200 and pLexA were the forward primer, 5′-ATCGGGATCCTTATGTTCTTGGTGAATTCGTTCTTGAAGG-3′, and the reverse primer, 5 5′-ACCGCTCGAGTCAGGAATACATAGTCAGCTGCAGCCAC-3′. PCR products were resolved with 1% agarose gels and visualized by ethidium bromide (1% solution, Fisher) staining. The resolved bands were then purified using a Qiaquick gel extraction kit (Qiagen, 28706). Purified PCR products, pCWX200 and pLexA were incubated with XhoI and BamHI (New England Biolabs) at 37 °C for 4 h in 1× buffer 3 supplemented with 1% BSA. The double-digested PCR products and plasmids were again purified with the Qiaquick gel extraction kit. To insert *Capn4* into pCWX200 and pLexA, the double-digested Capn4 PCR product was ligated with the double-digested pCWX200 or pLexA using the LigaFast Rapid DNA Ligation System (Promega, M8226). Successful insertions were confirmed by DNA sequencing (Applied Genomics Technology Center, Wayne State University). Bait plasmids were provided to ProteinLinks Inc. for the yeast two-hybrid assay. The candidates obtained *via* two-hybrid were identified by DNA sequencing (Applied Genomics Technology Center, Wayne State University).

### AlphaFold server predictive analysis of Capn4 candidate binding partners

To assess the likelihood of interaction between Capn4 and other candidate binding partners sequenced *via* yeast two-hybrid, we used the publicly available AlphaFold Server and input the amino acid sequences of Capn4 and the other 2-hybrid candidates. The likelihood that the model provides an accurate protein-protein interface is scored on a 0 to 1.0 scale using interface predicted template modeling (iPTM). The likelihood of the overall fold of the modeled complex is also scored on a 0 to 1.0 scale as predicted template modeling (pTM).

### Small-interfering RNA (siRNA) nucleofection

Wild-type MEFs were used to selectively silence *Bsg via* siRNA. The knockdown was generated through transient transfection with either control siRNA oligonucleotides or siRNA oligonucleotides targeting the *Bsg* gene using the siGENOME SMARTpool system (Dhamarcon). The siRNA oligonucleotides targeting the *Bsg* gene were: GAUUGGUUCUGGUUUAAGA, CAUCAGCAACCUUGACGUA, GCAAGUCCGAUGCAUCCUA,GGACAAGAAUGUACGCCAG. Nucleofection was performed using the Amaxa MEF2 Nucleofector Kit (Lonza) following the manufacturer’s suggested protocol. Briefly, MEF cells were trypsinized with 0.1% Trypsin-EDTA and collected by centrifuging at 2000 rpm for 5 min. Collected cells were then resuspended in an appropriate volume of the mixture, which included the MEF2 nucleofector solution, up to 5 μg of control siRNA or siRNA targeting the Bsg gene, and an empty vector of GFP and supplement 1, both provided with the kit. The total volume of the MEF2 Nucleofector solution, supplement 1 mixture, and siRNA was 100 μl and was transferred to an electroporation cuvette. The cuvette was then inserted into the Nucleofector II system (Amaxa), and the program MEF A-023 was run. 500 μl of RPMI-1640 medium (Sigma) was immediately added to the cuvette after the program was run to prevent cell damage. Nucleofected cells were then seeded according to the requirements of the following procedures. Inhibition of basigin expression reaches a maximum at 36 h after nucleofection.

### Protein extraction and Western blotting

Proteins were extracted from each cell line using triple-detergent lysis buffer (TDLB): pH 8, 50 mM Tris- HCl, 150 mM NaCl, 1% NP- 40, 0. 5% sodium deoxycholate, 0. 1% SDS, with added Protease Inhibitor Cocktail (Sigma) and HaltTM Phosphatase Inhibitor Cocktail (ThermoFisher). An 80% confluent 100 mm culture dish (NuncTM) was placed on ice and washed twice with ice-cold phosphate-buffered saline (PBS), then incubated for 25 min with 300 μl TDLB on ice. Lysed cells were collected into 1. 5 ml tubes using an ice-cold cell lifter and centrifuged at 13, 000 rpm for 10 min to remove cell debris. Protein concentration was determined using the Bio-Rad DC protein assay kit according to the manufacturer's instructions. Proteins were isolated from cell lines of MEFs, capn 4−/− cells, MEFs transfected with control siRNA, and MEFs transfected with siRNA targeting the Bsg gene. 20 micrograms of protein were loaded onto a 4 to 20% gradient Tris–HEPES–SDS precast polyacrylamide gel system (Pierce) and resolved at 100 V for 1 h. Proteins were then transferred onto an Immuno- Blot PVDF membrane (Bio- Rad) using a Trans-blot Turbo Transfer System (Bio- Rad) at 25 V for 15 min. After transfer, the membrane was blocked for 1 h with 5% milk in 1 × phosphate-buffered saline (PBS) – 0. 1% Tween (0. 1% PBS/T) for the Basigin p-antibody, or in 5% milk in 1 × Tris-buffered saline (TBS)- 0. 1% Tween (0. 1% TBS/T) for anti-actinin, anti- FLAG, and anti- HA antibodies. The membrane was then incubated overnight at 4 °C with primary antibodies under gentle agitation. The primary polyclonal or monoclonal anti-Basigin antibody (sc-9757, B-5, Santa Cruz) was diluted at 1:800 in 5% milk in 0.1% PBS/T. Anti-α-actinin antibody (A 5044, Sigma) and monoclonal anti-FLAG antibody (F 1804, Sigma) were diluted at 1:500 in 5% milk in 0.1% TBS/T. Monoclonal polyclonal anti- HA antibody (MMS- 101 P, Covance) was diluted at 1: 1000 in 5% milk in 0. 1% TBS/T. After washing three times for 20 min each with 0. 1% PBS/T, the membrane was incubated for 1 h at room temperature with secondary antibodies. HRP- linked anti-goat IgG (sc- 2020, Santa Cruz) at 1: 2000 dilution was used for the p-anti-basigin antibody, while HRP-linked anti-mouse antibody (ThermoFisher) at 1: 10, 0.1 dilution was used for anti-actinin, anti- FLAG, and anti- HA antibodies. For some blots, total protein was detected using No- Stain total protein labeling reagent (Invitrogen). After washing three times for 20 min each, detection was performed using ECL Plus Western Blotting Detection Reagents (Amersham), and visualization was done with an iBright 1500 (ThermoFisher).

### Cloning of CAPN4 and BSG and immunoprecipitation

Full-length *Capn4* was amplified by PCR from the pEGFP-capn4 plasmid and inserted into a pFLAG-CMV vector. The BSG gene, lacking the N-terminal 100 amino acids, was amplified from the pJG4-5-BSG vector obtained from the yeast two-hybrid assay and inserted into a pCDNA3 vector, together with an HA sequence. The primers used for amplification of *Capn4* were: forward primer 5′-CCCAAGCTTATGTTCTTGGTGAATTCG-3′ and reverse primer 5′-CCGGGATCCTCAGGAATACATAGTCAGCTGC-3′. The primers used for amplification of *Bsg* were forward primer 5′-CGCGGATCCATGGAAGGGCCACCCAGGATCAA-3′ and reverse primer 5′-CCGCTCGAGTCAGGTGGCGTTCCTCTGG-3′. Successful insertions were confirmed by sequencing (Applied Genomics Technology Center, Wayne State University).

293T cells were co-transfected with 10 μg of Flag-tagged Capn4 vector (full length) and 10 μg of HA-tagged Basigin containing vector. 20 h after the transfection, cells were harvested, and the immunoprecipitation assay was performed. Cells were lysed with ice-cold 1× lysis buffer (50 mM HEPES-NaOH, pH 7.5, 100 mM NaCl, 0.5% NP-40, 2.5 mM EDTA, 10% glycerol, 1 mM DTT) with Protease Inhibitor Cocktail (Sigma) and HaltTM Phosphatase Inhibitor Cocktail (Thermo) and then collected and pelleted by centrifugation. To 500 μg of cell lysate, 10 μg of anti-FLAG antibody or anti-HA antibody was added, and then the lysates were incubated for 1 h at 4 °C. 20 μl of Protein A/G PLUS Agarose (sc-2003, Santa Cruz) was added and incubated at 4 °C on a rocker platform overnight. The immunoprecipitate was collected by centrifugation, and the pellet was washed four times with 1× lysis buffer. After the final wash, the pellet was resuspended in 40 μl of electrophoresis sample buffer. The sample was boiled for 3 min and analyzed by SDS-PAGE with corresponding antibodies.

### Digestion of glycosyl units from basigin

Cell lysates were incubated with peptide-N-glycosidase F (PNGase F) (New England Biolabs) according to the manufacturer’s instructions. This involved incubating 9 μl of lysate with 1 μl of 10× Glycoprotein Denaturing Buffer at 95 °C for 10 min, followed by 5 min on ice. Samples were then received 2 uL 10× Reaction buffer, 2 uL 10% NP-40, 6 uL dH2O, and 1 uL PNGase F (final enzyme conc of 20 U/uL), and incubated at 37 °C for 2 h. Loading dye was added, and the samples were heated to 95 °C to stop the reaction. Samples were run on SDS-PAGE and blotted as above.

### Preparation of polyacrylamide substrates

A series of polyacrylamide substrates of different stiffnesses was prepared as described previously (53). Briefly, a flexible 75 μm × 22 mm polyacrylamide substrate was prepared in a cell culture chamber dish and embedded with 0.2 μm fluorescent microbeads. The acrylamide (acryl, Bio-rad) concentration was fixed at 5% while N, N-methylene-bis-acrylamide (bis, Bio-rad) varied from 0.04% to 0.1% to attain different stiffnesses of the substrates. The substrates were then coated with 5 μg/cm^2^ fibronectin (Sigma) at 4°C overnight, cross-linked with Sulfo-SANPAH (Thermo). Cells were seeded onto the substrates overnight before TFM or mechanosensing assays. The 5%/0.08% Acryl/Bis substrates (*E* = 1.41 kPa) were used in traction force microscopy (TFM), the 5%/0.1% Acryl/Bis substrates were used in the localized mechanosensing assay, and 5%/0.1% Acryl/Bis (hard) (*E* = 2.11 kPa) and 5%/0.04% Acryl/Bis (soft) (*E* = 0.41 kPa) substrates were used for the cell adhesion assay.

### Traction force microscopy

Flexible polyacrylamide substrates of 5%/0.08% Acryl/Bis coated with 5 μg/cm^2^ fibronectin were prepared as described above. Cells were seeded onto the flexible polyacrylamide substrates. Overnight cultures were imaged as described previously (53). Briefly, for a single cell, three images were taken under a 40× objective lens: a bright-field image of the cell, an image of the fluorescent beads with the cell on the substrate, and another image of the fluorescent beads after a microneedle had removed the cell, which served as the unstressed image. DIM (a program designed by Dr Yu-li Wang) was used to calculate bead displacement by comparing bead placement with and without the cell. The cell and nuclear boundaries were also outlined. These data, along with the Young's modulus, were used to generate and render traction stress values by using an algorithm courtesy of Dr Micah Dembo (Boston University) as described previously (31). Images of 11 to 15 cells for each cell line were collected. Data were analyzed by two-tailed Student *t* test.

### Mechanosensing assays

Flexible polyacrylamide substrates of 5/0.1% Acryl/Bis coated with fibronectin were prepared as described above. Cells were seeded onto the substrates and allowed to adhere overnight under regular cell culture conditions. As described previously (15), a cell was monitored for 10 min to track its migration trajectory, after which a blunted microneedle was pressed onto the substrate in the direction of the cell's migration, thereby exerting a pushing force on the cell. The pushing force would release the tension on the substrate, creating a localized soft spot. Images of cells were acquired using a 40× objective lens at 3-min intervals for 1 h to record the cells' migrating trajectories. If a cell responds to the pushing force applied by the needle by changing trajectory or rounding up, this is a positive response. If a cell continues to migrate along the same trajectory, it does not sense a change in the microenvironment and is recorded as nonresponsive. For each cell line, 6 to 10 cells were observed.

To explore the effect of global compliance, as opposed to the localized change in compliance tested above, on cellular morphology, polyacrylamide substrates of stiffness of 5%/0.1% Acryl/Bis (hard) and 5%/0.04% Acryl/Bis (soft) were made as described above. After solidification, the substrates were coated with 5 μg/cm^2^ fibronectin. Cells were coated onto the substrates and allowed to adhere overnight under standard cell culture conditions, after which images were recorded with a 10× objective. For nucleofected samples, cells were co-transfected with a GFP vector to indicate the transfected cells. The number of round cells was counted from zoomed images from six random fields for each cell line on both substrates with different stiffnesses. The cell numbers were plotted as box-and-whisker plots, and statistical analysis was done by a two-tailed student *t* test.

### Cell adhesion assay

A centrifugation assay was used to measure cell-substrate adhesiveness. This assay was performed using the method described by Guo *et al.* (32), with slight modifications. Briefly, a hole was drilled in an air-tight culture dish (Pall Corporation), and a coverslip was attached to the culture dish. 5%/0.08% Acryl/Bis substrates were prepared on coverslips as described above and then coated with 5 μg/cm^2^ fibronectin. 2.5 × 10ˆ4 cells were seeded onto fibronectin-coated substrates and allowed to adhere for 30 min at 37 °C. After incubation, the chambers were inverted and centrifuged at 1800*g* for 5 min. Ten random fields of cells were counted for each cell line right away after centrifugation. Percentages of cells before and after centrifugation are expressed in column graphs and standard deviation. Significance was determined by two-tailed student *t* test.

### Immunofluorescence

After being flamed, No. 1.5 glass coverslips (Fisher) were attached to chamber dishes. Then, they were coated with 5 μg/cm^2^ fibronectin (Sigma) at 4 °C overnight and blocked with 1% BSA in PBS at 4 °C overnight. Cells were seeded onto coverslips and allowed to attach overnight under incubation at 37 °C with 5% CO2 in a humidified cell culture incubator. The cells were then fixed and permeabilized with the following steps: firstly, incubate for 10 min with 2.5% paraformaldehyde in 1× PBS at 37 °C; then incubate with 2.5% paraformaldehyde in 1× PBS with 0.1% Triton X-100 at 37 °C; followed by incubation for 5 min with 0.5 mg/ml NaBH_4_ solution. After fixation and permeabilization, cells were blocked with 5% BSA in PBS for 1 h at room temperature. Then they were incubated with basigin antibody (sc-9757, Santa Cruz) at a 1:250 dilution for 3 h at room temperature. Following three 15-min washes, Alexa Fluor 546 anti-goat secondary antibody was added at a 1:500 dilution in 5% BSA for 1 h at room temperature. For focal adhesion immunostaining, fixed cells were incubated with vinculin antibody (Sigma, V4505) at a 1:200 dilution for 3 h at room temperature. Following three 15-min washes, Alexa Fluor 568 anti-mouse secondary was added at a 1:500 dilution in 5% BSA for 1 h at room temperature. After the final washes (3 × 15 min each), mounting media (pH = 7.8, 0.1% PPD, 1× PBS, 50% glycerol, 30% Q-H_2_O) was added. Images were taken with appropriate filters for GFP signals and AF 568 signals. Immunostained adhesions were analyzed by ImageJ and manual counts and plotted as box and whisker plots.

#### Cell migration assay

After being flamed, No. 1.5 square glass coverslips (Fisher) were attached to chamber dishes and coated with 5 μg/cm^2^ fibronectin (Sigma) diluted in 50 mM HEPES at 4˚C overnight. Cells were then seeded onto the coverslips and allowed to attach overnight under incubation at 37 °C with 5% CO2 in a humidified cell culture incubator. Images were acquired to describe the migration trajectory of a single cell over 2 h at 2-min intervals using a 40× objective lens. All the collected images for a single cell were imported into the custom-built dynamic image analysis system (DIM, Y-L. Wang) to calculate the linear speed and persistence of each cell. Ten cells were observed for each cell line and plotted as box and whisker, and significance determined by two-tailed student *t* test.

#### Microscopy

Images of all experiments described above were acquired using an Olympus IX81 ZDC inverted microscope equipped with a custom-built stage incubator to maintain cells at 37 °C under 5% CO2 for live-cell imaging. A SPOT Boost EM-CCD-BT2000 back-thinned camera (Diagnostic Instruments Inc) was also used. The camera was run by IPLab software (BD Biosciences). Adhesions were imaged on an Echo Revolve2-K3-2134 (Discover Echo Inc) fluorescent microscope adjusted for inverted imaging.

### GEPIA2-guided pan-cancer analysis of CAPN4 and Bsg

Given the propensity for CAPN4 and Bsg overexpression in tumor progression, we examined their correlation across various tumor types of interest. The GEPIA2 (Gene Expression Profiling Interactive Analysis) web-based platform serves as a centralized source of GTEx and TCGA gene expression data (44). Using the Correlation Analysis function from GEPIA2, Pearson’s correlation coefficient was determined for 33 TCGA tumor tissues, and we identified several cancers where CAPN4 and Bsg expression were positively correlated (Fig. 6). Statistical significance of each correlation’s strength was also determined by GEPIA2 using a *t* test for Pearson’s correlation coefficient.

## Data availability

All data are contained within the manuscript; if further information is necessary, it may be requested from the corresponding author, Karen Beningo, beningo@wayne.edu.

## Supporting information

This article contains supporting information.

## Declaration of Generative AI and AI-Assisted Technologies in the Writing Process

Grammarly was used for spell check and to optimize sentence structure in this manuscript. All concepts, data, and interpretations were produced by the authors.

## Conflict of interest

The authors declare that they have no conflicts of interest with the contents of this article. The author is an Editorial Board Member/Editor-in-Chief/Associate Editor/Guest Editor for this journal and was not involved in the editorial review or the decision to publish this article.
