# Supplementary material for: Basigin (CD147) and calpain 4 (CapnS1) are partners in the generation of traction force but not in mechanosensing
Source: J Biol Chem. 2026 May 18;302(7):113170. doi: 10.1016/j.jbc.2026.113170 (PMC13279014; doi:10.1016/j.jbc.2026.113170)
Supplement: Supporting Information [file mmc1.pptx]

## Slide 1
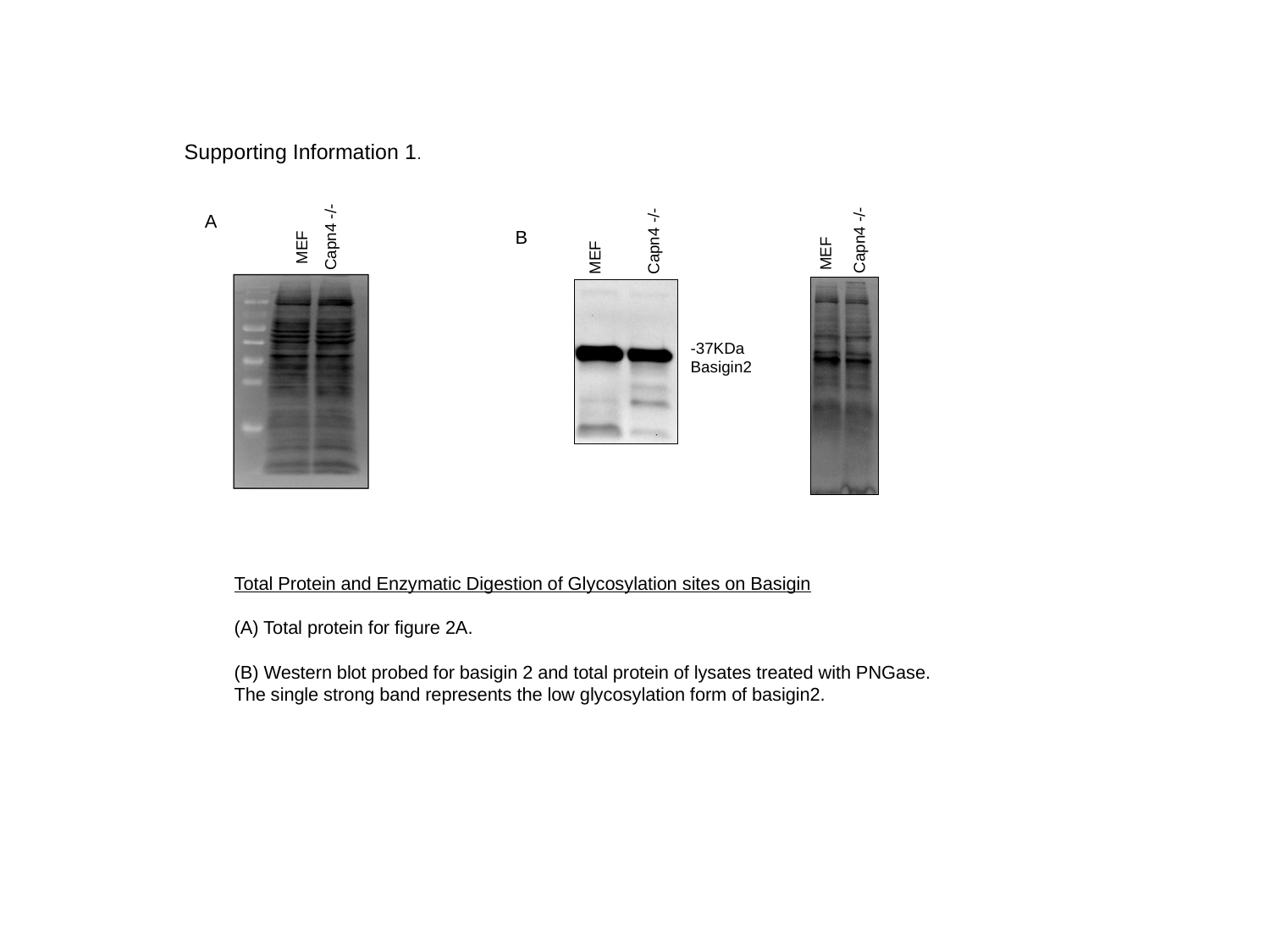

Supporting Information 1.
A
B
Capn4 -/-
Capn4 -/-
Capn4 -/-
MEF
MEF
MEF
-37KDa
Basigin2
Total Protein and Enzymatic Digestion of Glycosylation sites on Basigin
(A) Total protein for figure 2A.
(B) Western blot probed for basigin 2 and total protein of lysates treated with PNGase.
The single strong band represents the low glycosylation form of basigin2.

## Slide 2
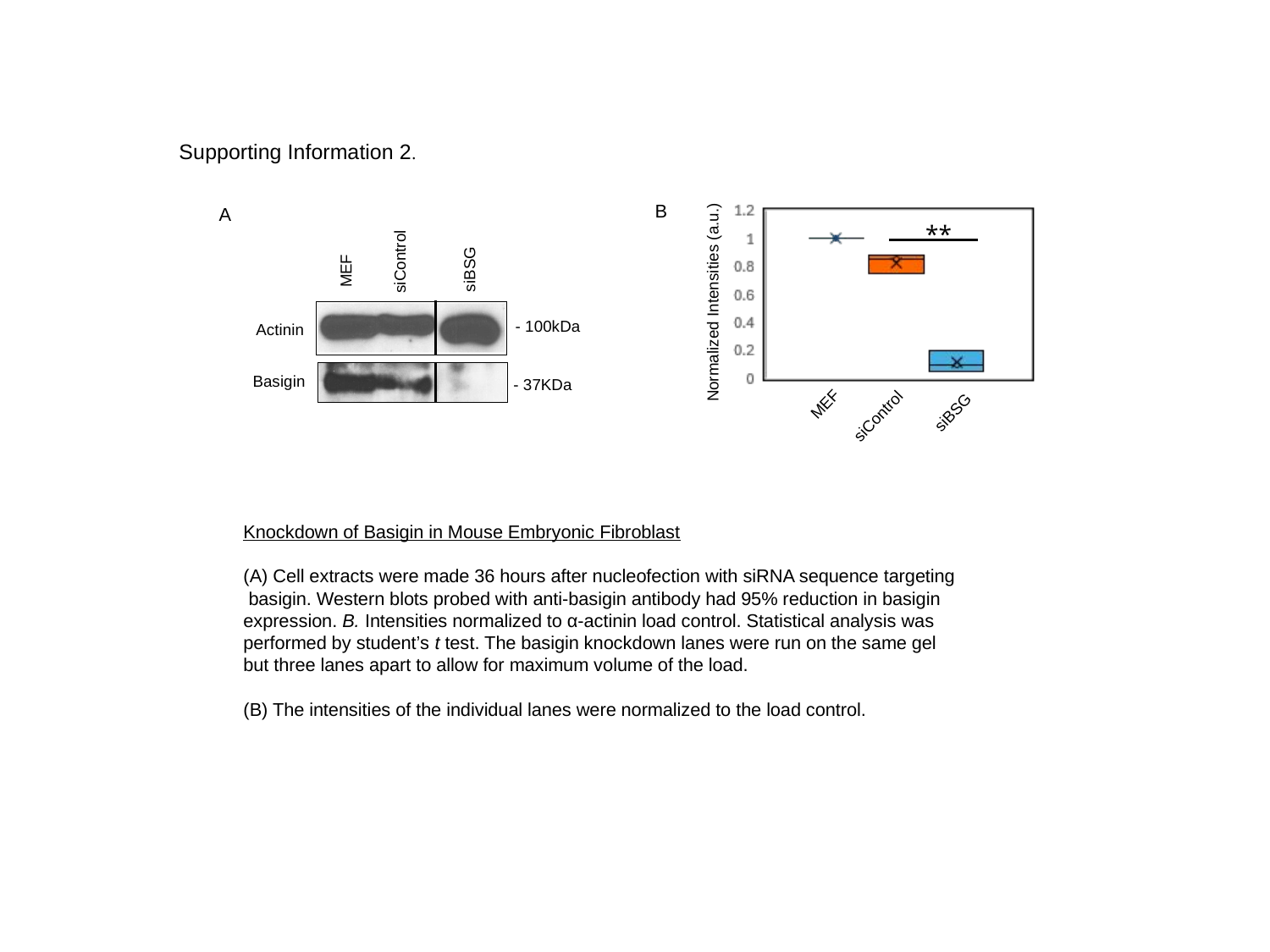

Supporting Information 2.
A
siControl
siBSG
MEF
Actinin
Basigin
B
MEF
siBSG
 siControl
**
Normalized Intensities (a.u.)
- 100kDa
- 37KDa
*
Knockdown of Basigin in Mouse Embryonic Fibroblast
(A) Cell extracts were made 36 hours after nucleofection with siRNA sequence targeting
 basigin. Western blots probed with anti-basigin antibody had 95% reduction in basigin
expression. B. Intensities normalized to α-actinin load control. Statistical analysis was
performed by student’s t test. The basigin knockdown lanes were run on the same gel
but three lanes apart to allow for maximum volume of the load.
(B) The intensities of the individual lanes were normalized to the load control.

## Slide 3
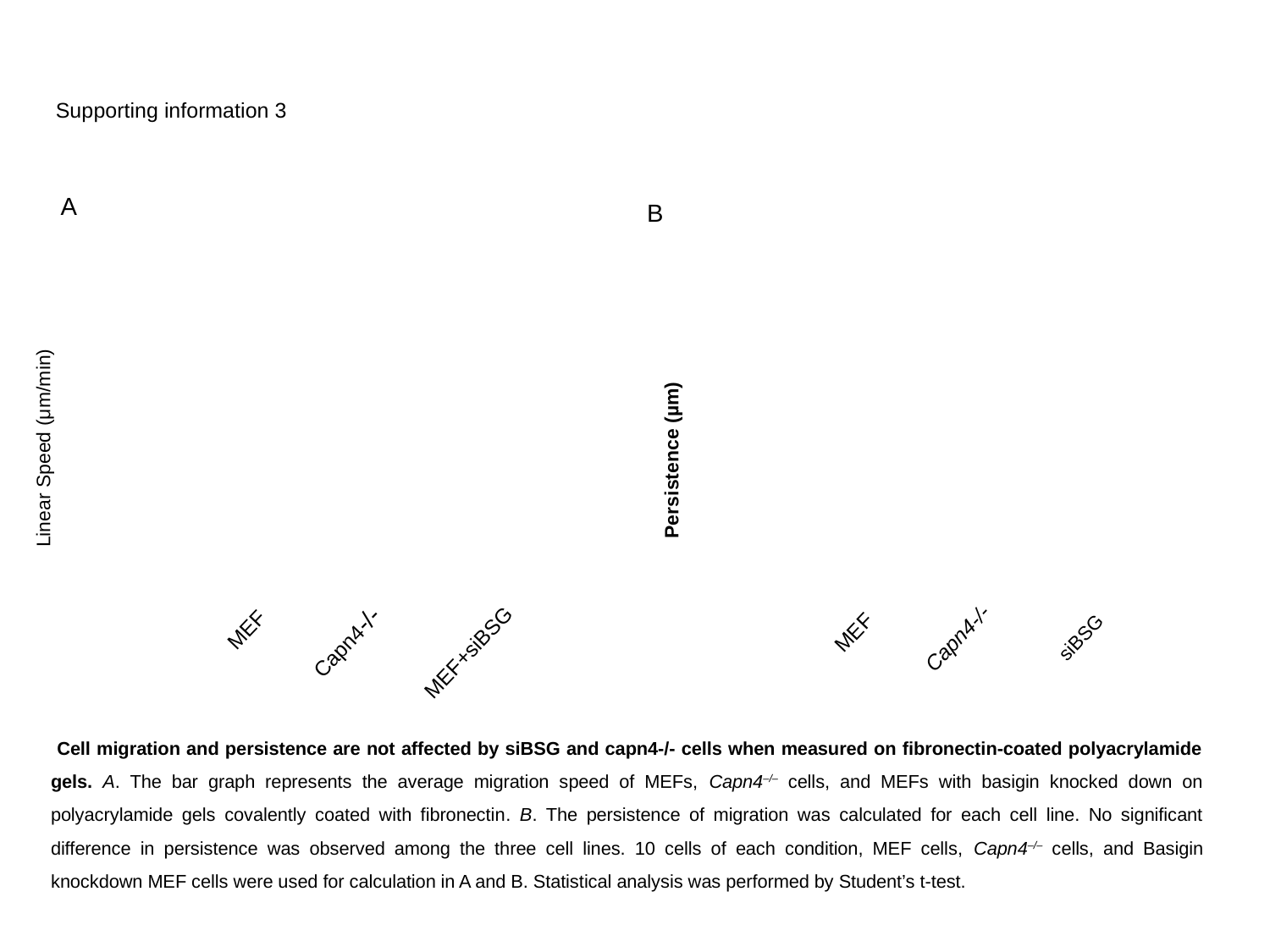

Supporting information 3
A
Linear Speed (μm/min)
MEF
Capn4-/-/-
MEF+siBSG
B
Persistence (µm)
MEF
Capn4-/-
MEF +siBSG
 Cell migration and persistence are not affected by siBSG and capn4-/- cells when measured on fibronectin-coated polyacrylamide gels. A. The bar graph represents the average migration speed of MEFs, Capn4–/– cells, and MEFs with basigin knocked down on polyacrylamide gels covalently coated with fibronectin. B. The persistence of migration was calculated for each cell line. No significant difference in persistence was observed among the three cell lines. 10 cells of each condition, MEF cells, Capn4–/– cells, and Basigin knockdown MEF cells were used for calculation in A and B. Statistical analysis was performed by Student’s t-test.
